# A new arthroscopic repair technique for triangular fibrocartilage complex using an intracapsular suture: an outside-in transfer all-inside repair

**DOI:** 10.1186/s13018-023-04386-0

**Published:** 2023-11-24

**Authors:** Jiasong Zhao, Yanming Lin, Lang Li, Yong Huang

**Affiliations:** 1 Hospital of Chengdu University of Traditional Chinese Medicine, Chengdu, China; 2 Hospital of Chengdu Office of People’s Government of Tibetan. Autonomous Region, Chengdu, China

**Keywords:** Peripheral triangular fibrocartilage complex, All-inside, Outside-in, Arthroscopic repair

## Abstract

**Background:**

Arthroscopic repair is a promising, minimally invasive surgical technique for patients with Palmer type 1B peripheral triangular fibrocartilage complex (TFCC) tears. Although several arthroscopic techniques are effective for repairing Palmer type 1B TFCC tears, some shortcomings remain. So, we report an arthroscopic repair technique for the treatment of Palmer type 1B Atzei class 1 TFCC tears using an intracapsular suture: an outside-in transfer all-inside repair.

**Methods:**

A retrospective analysis of 38 Palmer type 1B TFCC injury patients admitted to our hospital were randomly divided into 2 groups. The group A was sutured from the outside to the inside, with a total of 21 cases; the group B was sutured with the new arthroscopic repair technique, with a total of 17 cases. Observe and compare the VAS scores and modified Mayo wrist function scores of all patients before 3, and 6 months after the operation and evaluate the incidence of thread knots in patients with different treatment methods. The methodology was performed an arthroscopic intracapsular suture using an outside-in transfer, all-inside repair technique, which is a modified method of the outside-in and all-inside technique using the needle of a 10-mL sterile syringe, for Palmer type 1B TFCC tears. A No. 2 polydioxanone suture was threaded through the needle and entered the wrist joint. Next, the needle was withdrawn carefully along the suture to the proximal tear ulnar surface of the TFCC and penetrated the TFCC, exiting the articular cavity surface of the ulnar side of the torn TFCC. Finally, arthroscopic knotting was performed.

**Results:**

This new treatment was as effective as the previously arthroscopic techniques and had the advantages of no additional incision and decreased risk of operation-related complications. The incidence of thread knots in the group A (28.57%) was significantly higher than that in the group B (0%), and the difference was statistically significant (*P* = 0.024). There was no significant difference in VAS score and modified Mayo wrist function scores between the two groups (*P* > 0.05).

**Conclusions:**

The outside-in transfer, the all-inside repair technique is suitable for Palmer type 1B Atzei class 1 TFCC tears. We recommend this technique as a useful alternative to the conventional methods of repairing Palmer type 1B TFCC tears.

**Supplementary Information:**

The online version contains supplementary material available at 10.1186/s13018-023-04386-0.

## Introduction

The triangular fibrocartilage complex (TFCC) is the most important stabilizer of the distal radioulnar joint and act as a shock absorber across the ulnocarpal joint [1, 2]. The TFCC is composed of the fibrocartilaginous disk, the dorsal, and palmar ligaments spanning across the radius and ulna, the ulnocarpal ligaments, a meniscal homolog, and the subsheath of the ulnar extensor of the wrist, whose critical stable component inserts directly in the ulna, either deep into the fovea (ligamentum subcruentum) or at the base of the styloid [1]. Traumatic injury, including axial loading of an ulnar deviated wrist and the disruption of normal ulnar variance, to the TFCC, is a frequent cause of ulnar-sided pain and wrist disability [3].

When movements of wrist rotation occur, TFCC injury usually leads to a patient complaint of activity-related ulnar-sided wrist pain, which may also be accompanied by grip weakness, instability, clicking at presentation, and weakness in pronation and supination [4]. Imaging examination, including radiography, Magnetic resonance imaging (MRI), and MRI with arthrography, is necessary to evaluate TFCC injury [5, 6]. The recent developments of MRI, including the high-resolution two-dimensional/three-dimensional sequences and 3-T field strength, may improve the detection of TFCC injuries that are difficult to evaluate on routine sequences [7]. Arthroscopy is the most accurate means by which to diagnose TFCC injury irrespective of location [8].

The Palmer classification system, the most widely used scheme, classifies TFCC injuries into type I (traumatic tear) and type II (degenerative tear) according to the location and chronicity of the tear [9]. Palmer type I B tears represent traumatic peripheral tears of the TFCC from its ulnar insertion and tend to be the most amenable to surgical repair when conservative treatment is ineffective [9]. A treatment-oriented classification system especially focusing on Palmer type 1B was proposed by Atzei, which subdivides Palmer type 1B peripheral tears into five types as follows: class l repairable distal tears; class 2, repairable complete tears; class 3, repairable proximal tears; class 4, non-repairable tears; and class 5, tears associated with distal radioulnar joint (DRUJ) arthritis [10]. Atzei advocated that class 1 tears should be sutured and that class 2 and 3 tears are associated with DRUJ instability and require TFCC reattachment to the fovea [11]. Class 1 tears were currently repaired using arthroscopy [11–15]. Arthroscopic TFCC foveal repair techniques for class 2 and 3 tears have already been introduced [10, 16–18]. However, some complications such as subcutaneous suture knotting and injury of the extensor carpi ulnaris tendon or sensory branch of the ulnar nerve still occur with the arthroscopic technique for the repair of Palmer type 1B TFCC tears. The goal of surgeons has always been to reduce the risk of surgery and complications by developing new methods that can increase TFCC tear healing and reduce complications.

Here, we report an arthroscopic repair technique for the treatment of Palmer type 1B Atzei class 1 TFCC tears. This technique could be used to suture the TFCC tissue without the capsule and subcutaneous tissue and could preserve the normal biomechanics of the meniscus during motion and reduce complications.

## Methods

**Fig. 1:**
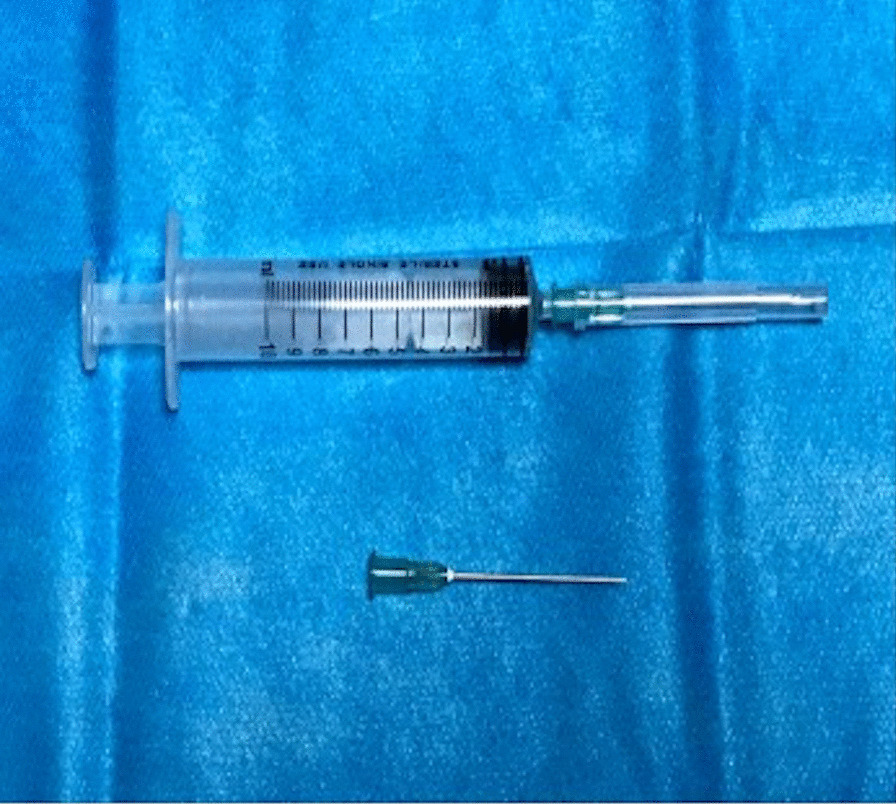
A needle of 10 ml sterile syringe

**Fig. 2:**
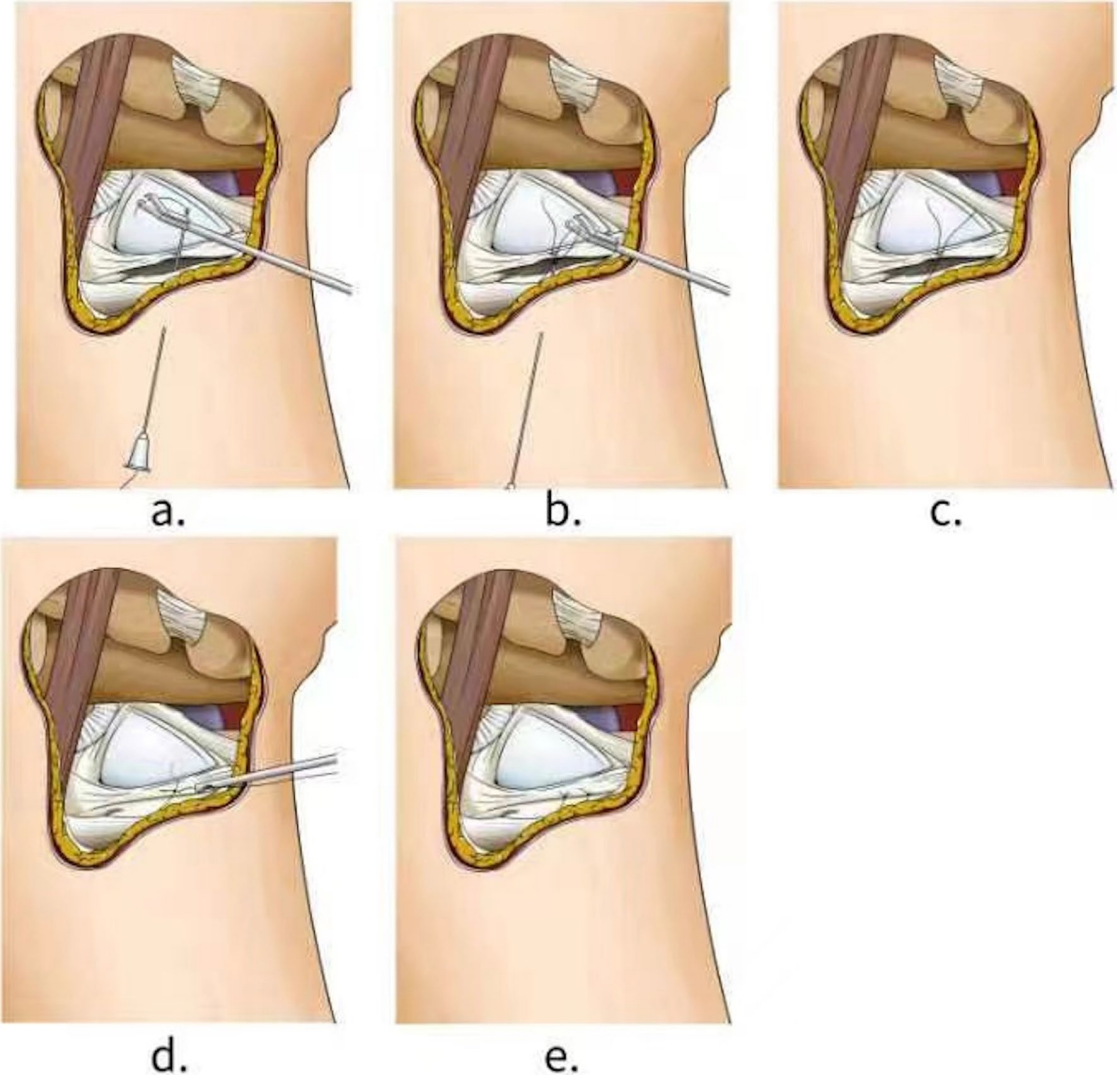
Suture method using a needle and suture. **A** The needle with 2 PDS suture penetrates the lower surface of the TFCC, and one limb of the PDS suture is pulled out with a grasper from the 6R portal. **B** The needle is withdrawn to the subcutaneous. **C** Adjust the angle, the needle penetrates the upper surface of the TFCC, another limb of the PDS suture is pulled out with a grasper from the 6R portal (the two limbs of the suture were retrieved through the same arthroscopy portal using a grasper and formed a sliding knot inside the joint. **D** Tennessee Knots and then additional knots were made to secure the knot. The suture strands were cut by knot cutter and then the knot was pushed to the upper surface of the TFCC

This retrospective study was conducted in Hospital of Chengdu University of Traditional Chinese Medicine. 38 Palmer type 1B TFCC injury patients between August 2017 and June 2020 were enrolled in this study cohort. The patient was positioned for standard wrist arthroscopy using a standard traction apparatus. To probe and evaluate TFCC tears, the 3–4, 4R, and 6R portals were routinely performed. This new repair technique for subtype Palmer type 1B Atzei class 1 TFCC tears requires the needle of a 10-mL sterile syringe, an arthroscopic retriever, a knot pusher, and suture material. A 2.7-mm 30° arthroscope was used to the visualize TFCC tear via 3 or 4 portals, and the probe was inserted through the 6R portal to check and assess the tear parameter of the TFCC, including the site, size, pattern, stability, tissue quality, and associated pathology in the wrist joint. Before sewing, the tear site of the TFCC was debrided with a 2.9-mm full-radius motorized shaver until bleeding to remove the proliferative synovial tissue and encourage healing through the 6R portal. Then, the group A was sutured from the outside to the inside, the group B was sutured with the outside-in transfer, all-inside repair technique to repair the TFCC tear (Additional file 1). First, on the skin near the tear ulnar site of the TFCC, the needle of a 10-mL sterile syringe penetrated the skin, subcutaneous tissue, articular capsule, and finally, the proximal tear radial surface of the TFCC, and then exited the articular cavity surface of the radial side of the torn TFCC (Fig. 1). Second, a No. 2 polydioxanone (PDS) suture was threaded through the needle of a 10-mL sterile syringe and inserted in the wrist joint. Then, the suture tip was retrieved through the 6R portal with a grasper (Fig. 2). Third, the needle was withdrawn carefully along the suture to the proximal tear on the ulnar surface of the TFCC, avoiding the suture while retreating the needle at the same time. After adjusting the needle puncture direction, the needle was reinserted upward and penetrated through the proximal tear of the ulnar surface of the TFCC, exiting the articular cavity surface of ulnar side of the torn TFCC. The procedure was carefully performed to avoid cutting the suture with the tip of needle. The suture end was folded in the tip of needle and pulled out with a grasper through the 6R portal. Here the outside-in repair technology was successfully converted to an all-inside knotting technology. Next, after confirming the tear site of the TFCC penetrated by both limbs of the suture, the suture was slowly tensioned to obtain fine reduction of the torn TFCC under arthroscopic visualization. Furthermore, the Samsung Medical Center sliding knot technique was used to form the knot, which was positioned on the synovial side to avoid articular cartilage erosion of the scaphoid and lunate bone during wrist motion. After the knot was performed, the tension was carefully checked with a probe via arthroscopic visualization. A second knot or more was also made in a similar way if it was necessary to acquire a stable TFCC repair. Finally, to assess the stability of the suture, the full range of repeated wrist motion must be finished.

## Results

A total of 38 Palmer type 1B TFCC injury patients, including 21 males and 17 females, were enrolled in this retrospective study. Patient demographics of these TFCC patients on admission are shown in Table 1. 17 cases in the group B, with an average age of 31.88 ± 7.03 years, and an average operation time of 52.88 ± 4.78 min. 21 cases in the group A, with an average age of 29.29 ± 8.30 years, and an average operation time of 52.19 ± 4.88 min (Table 1).

**Table 1:** Patient demographics associated with TFCC on admission

Group	Case	Age (years, $\overline{x}$ ± *s*)	Gender		Site		Operation duration (min)
			Male	Female	Left	Right	
B	17	31.88 ± 7.03	9	8	5	12	52.88 ± 4.78
A	21	29.29 ± 8.30	12	9	7	14	52.19 ± 4.88
Statistic	–	1.026	–		–		0.427
*P* value	–	0.312	1.000		1.000		0.672

The results showed that the group B had more suture reaction than the group A in Table 2 (*P* = 0.024). There was no significant difference in VAS score and mayo score before operation, 3 and 6 months after between the two groups (*P* > 0.05) (Table 2).

**Table 2:** Comparison of curative effects between the two groups

Group	Case	Thread knots	VAS scores			Mayo scores		
			Before	3 months after	6 months after	Before	3 months after	6 months after
B	17	0	5.12 ± 1.50	1.82 ± 1.07	0.18 ± 0.39	59.59 ± 4.29	78.76 ± 4.55	91.12 ± 3.44
A	21	6	5.29 ± 1.06	1.67 ± 1.11	0.43 ± 0.68	59.09 ± 4.89	79.14 ± 5.22	89.62 ± 3.94
Statistic	–	–	− 0.406	0.737	1.184	0.358	− 0.235	1.232
*P* value	–	0.024	0.687	0.461	0.236	0.723	0.816	0.226

## Discussion

In this study, the incidence of thread knots in the group A (28.57%) was significantly higher than that in the group B (0%), and the difference was statistically significant (*P* = 0.024). There was no significant difference in VAS score and modified Mayo wrist function scores between the two groups (*P* > 0.05). This new treatment was as effective as the previously arthroscopic techniques and had the advantages of no additional incision and decreased risk of operation-related complications [3, 19].

**Fig. 3:**
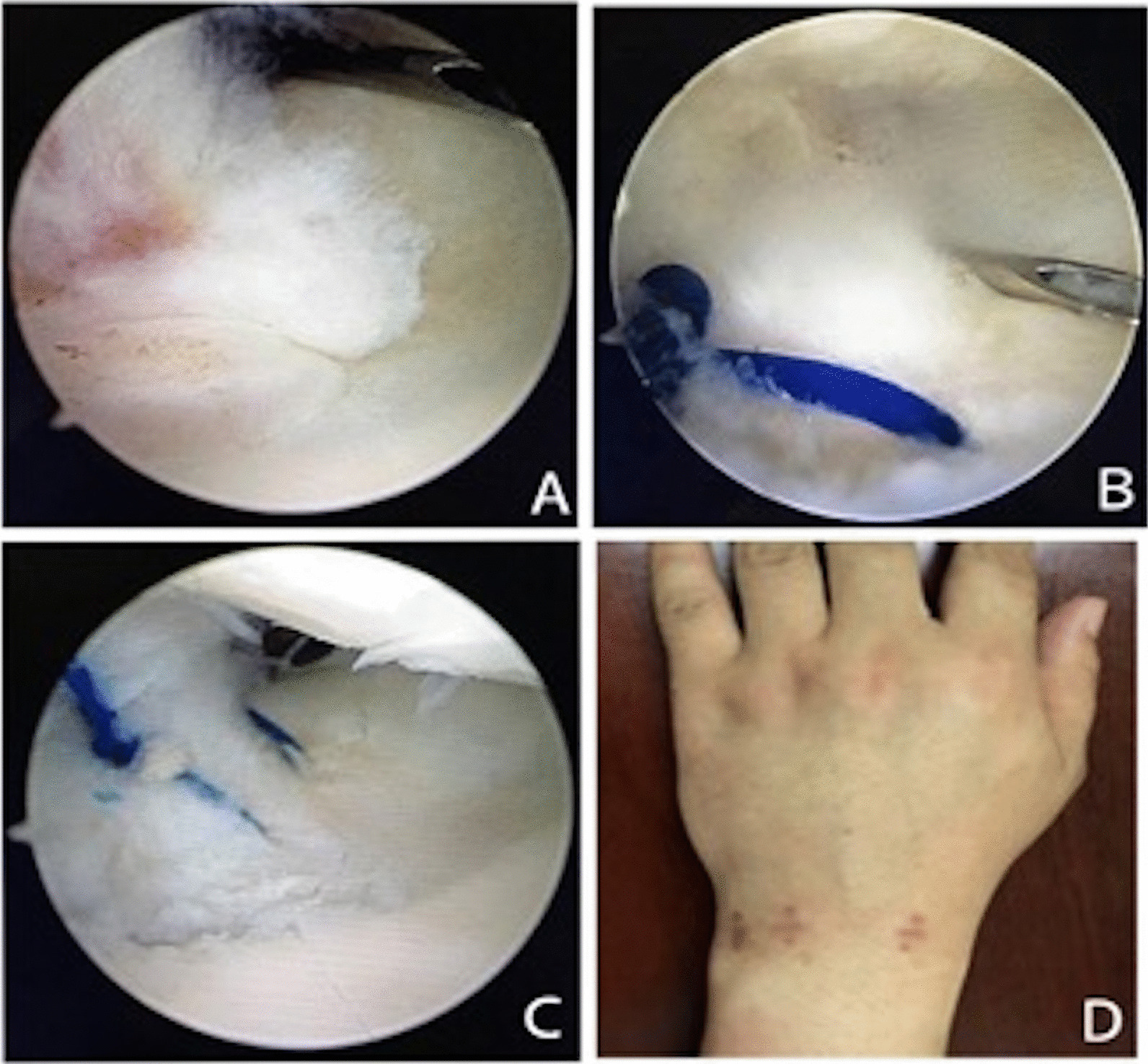
One patient with Palmer type 1B TFCC tear was operated using the outside-in transfer, all-inside technique. **A** Arthroscopic examination revealed the TFCC injury, Palmer type I B, superficial injury. **B** The needle with PDS suture penetrates the lower surface of the TFCC. **C** Repair of injured TFCC using a needle and suture. **D** The knots don't cause an inflammatory reaction after the operation

A new arthroscopic intracapsular suture repair technique called outside-in transfer, the all-inside repair was used for Palmer type 1B Atzei class 1 TFCC tears in this study. This new method is a modification of the technique for meniscal tears by Wang in 2019 [20]. This repair technique using arthroscopy provides several advantages to other reported repair techniques. First, it is easy to accomplish because it first uses the outside-in technique and then transfer to using the needle of 10-mL sterile syringe, which is cheaper than other instruments. Second, it allows the use of a vertical mattress suture, which is useful for the alignment of the edge of TFCC tear and is easier to heal. Third, the suture knots of this outside-in transfer, all-inside technique can be performed without an additional skin incision and placed inside the joint instead of subcutaneously to avoid irritating the skin and injuring the dorsal branch of the ulnar nerve and extensor carpi ulnaris tendon (Fig. 3).

Several suture techniques are available for Palmer type 1B Atzei class 1 TFCC tears. The currently used techniques of arthroscopic repair for class 1 tears include the inside-out, outside-in, or all-arthroscopic technique [11–15]. An outside-in technique using 2 needles guiding 2 sutures to repair the TFCC was first described by Zachee et al. [21]. Both Trumble et al. and Skie et al. advocated an inside-out technique for Palmer type 1B TFCC repair using 2–0 meniscal repair sutures [22, 23]. Although these techniques have been modified, they require an extra incision to tie the sutures subcutaneously, which confers a risk of injury to the extensor carpi ulnaris tendon or the sensory branch of the ulnar nerve. Furthermore, the suture knot lies subcutaneously, causing skin problems and even septic arthritis [24–27]. A study of an all-arthroscopic repair technique for TFCC with the outside-in technique in fresh-frozen cadaveric wrists showed that the PDS knot was 4.6 mm from the dorsal branch of the ulnar nerve, which may be injured by the knot, and injured an extensor carpi ulnaris tendon [24]. Another cadaver study showed that the mean minimum distance between the suture and the dorsal branch of the ulnar nerve was 1.9 mm in the inside-out technique [28]. In a previous study, Bayoumy reported that 37 patients with TFCC tears were treated with the arthroscopic outside-in repair technique, in which two patients showed complications, including dorsal ulnar nerve neurapraxia in one patient and weakness in extension of the little finger in the other patient [29]. The goal of surgeons has always been to reduce the risk of surgery and complications as much as possible by developing a new method that can increase TFCC tear healing and reduce complications. In our consecutive treatment series, none of the patients had complications such as skin problems, injury of the dorsal branch of the ulnar nerve, and injury of the extensor carpi ulnaris tendon (Additional file 1).

As in knee arthroscopy, an all-inside technique should be fast and safe to use and avoid the disadvantages of the other techniques. A study by Conca et al. in 2003 described an all-inside repair technique for Palmer type 1B TFCC tears using a small suture hook and three portals [30]. Böhringer et al. used a meniscus fastener fixation system to repair Palmer 1B TFCC tears [27]. A novel all-inside approach for Palmer type 1B TFCC tears with a spinal needle and no additional incision was introduced and described by Lee et al. [14]. Kuremsky et al. [31] assessed the safety of an all-inside arthroscopic TFCC repair technique in 13 above-the-elbow human cadaver specimens. The results of this study showed that the all-inside technique was safe in terms of proximity to important structures. However, the technique had a significant drawback in that the intra-articular working space in the wrist was so narrow that the range of manipulation with the suture devices through the portal was restricted. Our technique is simpler than the other all-inside techniques, although one portal without special equipment is required.

A new arthroscopic intracapsular suture repair technique called outside-in transfer, all-inside repair was used for Palmer type 1B Atzei class 1 TFCC tears in this study. This new method is a modification of the technique for meniscal tears introduced by Wang et al. [20]. This repair technique using arthroscopy provides several advantages to other reported repair techniques. First, this technique is easy to accomplish because, first, it uses the outside-in technique and then transfer to using the needle of 10-mL sterile syringe, which is cheaper than other instruments. Second, it allows the use of a vertical mattress suture, which is useful for the alignment of the edge of the TFCC tear and faster tear healing. Third, the suture knots of this outside-in transfer, all-inside technique can be performed without an additional skin incision and placed inside the joint instead of subcutaneously to avoid irritating the skin and injuring the dorsal branch of the ulnar nerve and extensor carpi ulnaris tendon. Finally, this technique is easier to perform than other techniques.

## Conclusion

In conclusion, the outside-in transfer, the all-inside repair technique is suitable for Palmer type 1B Atzei class 1 TFCC tears. We recommend this technique as a useful alternative to the conventional methods of repairing Palmer type 1B TFCC tears.

### Supplementary Information

**Additional file 1**. The video of the technique called outside-in transfer, all-inside repair.

## Data Availability

All data analyzed in this study has been provided in the manuscript.
